# Assigning mutational signatures to individual samples and individual somatic mutations with SigProfilerAssignment

**DOI:** 10.1093/bioinformatics/btad756

**Published:** 2023-12-14

**Authors:** Marcos Díaz-Gay, Raviteja Vangara, Mark Barnes, Xi Wang, S M Ashiqul Islam, Ian Vermes, Stephen Duke, Nithish Bharadhwaj Narasimman, Ting Yang, Zichen Jiang, Sarah Moody, Sergey Senkin, Paul Brennan, Michael R Stratton, Ludmil B Alexandrov

**Affiliations:** Department of Cellular and Molecular Medicine, UC San Diego, La Jolla, CA 92093, United States; Department of Bioengineering, UC San Diego, La Jolla, CA 92093, United States; Moores Cancer Center, UC San Diego, La Jolla, CA 92037, United States; Department of Cellular and Molecular Medicine, UC San Diego, La Jolla, CA 92093, United States; Department of Bioengineering, UC San Diego, La Jolla, CA 92093, United States; Moores Cancer Center, UC San Diego, La Jolla, CA 92037, United States; Department of Cellular and Molecular Medicine, UC San Diego, La Jolla, CA 92093, United States; Department of Bioengineering, UC San Diego, La Jolla, CA 92093, United States; Moores Cancer Center, UC San Diego, La Jolla, CA 92037, United States; Department of Cellular and Molecular Medicine, UC San Diego, La Jolla, CA 92093, United States; Department of Bioengineering, UC San Diego, La Jolla, CA 92093, United States; Moores Cancer Center, UC San Diego, La Jolla, CA 92037, United States; Department of Cellular and Molecular Medicine, UC San Diego, La Jolla, CA 92093, United States; Department of Bioengineering, UC San Diego, La Jolla, CA 92093, United States; Moores Cancer Center, UC San Diego, La Jolla, CA 92037, United States; COSMIC, Wellcome Sanger Institute, Hinxton, Cambridgeshire CB10 1SA, United Kingdom; COSMIC, Wellcome Sanger Institute, Hinxton, Cambridgeshire CB10 1SA, United Kingdom; Department of Cellular and Molecular Medicine, UC San Diego, La Jolla, CA 92093, United States; Department of Bioengineering, UC San Diego, La Jolla, CA 92093, United States; Moores Cancer Center, UC San Diego, La Jolla, CA 92037, United States; Department of Cellular and Molecular Medicine, UC San Diego, La Jolla, CA 92093, United States; Department of Bioengineering, UC San Diego, La Jolla, CA 92093, United States; Moores Cancer Center, UC San Diego, La Jolla, CA 92037, United States; Department of Cellular and Molecular Medicine, UC San Diego, La Jolla, CA 92093, United States; Department of Bioengineering, UC San Diego, La Jolla, CA 92093, United States; Moores Cancer Center, UC San Diego, La Jolla, CA 92037, United States; Cancer, Ageing and Somatic Mutation, Wellcome Sanger Institute, Wellcome Genome Campus, Cambridge CB10 1SA, United Kingdom; Genetic Epidemiology Group, International Agency for Research on Cancer, 69372 Lyon, France; Genetic Epidemiology Group, International Agency for Research on Cancer, 69372 Lyon, France; Cancer, Ageing and Somatic Mutation, Wellcome Sanger Institute, Wellcome Genome Campus, Cambridge CB10 1SA, United Kingdom; Department of Cellular and Molecular Medicine, UC San Diego, La Jolla, CA 92093, United States; Department of Bioengineering, UC San Diego, La Jolla, CA 92093, United States; Moores Cancer Center, UC San Diego, La Jolla, CA 92037, United States

## Abstract

**Motivation:**

Analysis of mutational signatures is a powerful approach for understanding the mutagenic processes that have shaped the evolution of a cancer genome. To evaluate the mutational signatures operative in a cancer genome, one first needs to quantify their activities by estimating the number of mutations imprinted by each signature.

**Results:**

Here we present SigProfilerAssignment, a desktop and an online computational framework for assigning all types of mutational signatures to individual samples. SigProfilerAssignment is the first tool that allows both analysis of copy-number signatures and probabilistic assignment of signatures to individual somatic mutations. As its computational engine, the tool uses a custom implementation of the forward stagewise algorithm for sparse regression and nonnegative least squares for numerical optimization. Analysis of 2700 synthetic cancer genomes with and without noise demonstrates that SigProfilerAssignment outperforms four commonly used approaches for assigning mutational signatures.

**Availability and implementation:**

SigProfilerAssignment is available under the BSD 2-clause license at https://github.com/AlexandrovLab/SigProfilerAssignment with a web implementation at https://cancer.sanger.ac.uk/signatures/assignment/.

## 1 Introduction

Somatic mutations accumulate in the genomes of all cells of the human body (Stratton *et al.* 2009, Martincorena and Campbell 2015). These mutations arise from different mutational processes, with each process generating a characteristic pattern of mutations, known as a *mutational signature* (Alexandrov *et al.* 2013). By leveraging the vast amounts of high-throughput DNA sequencing data generated over the past two decades, distinct mutational signatures have been elucidated from various cancer types (Alexandrov *et al.* 2020, Degasperi *et al.* 2022) and normal somatic tissues (Lee-Six *et al.* 2019, Lawson *et al.* 2020, Olafsson *et al.* 2020, Yoshida *et al.* 2020). Sets of mutation-type specific reference signatures have been developed and deposited in the Catalogue of Somatic Mutations in Cancer (COSMIC) database (Tate *et al.* 2019, Alexandrov *et al.* 2020), including signatures of single base substitutions (SBSs), doublet base substitutions (DBSs), small insertions and deletions (IDs), and copy number alterations (CNs).

There are at least two distinct approaches for analyzing mutational signatures. *De novo* extraction is an unsupervised machine learning approach that allows identifying the patterns of known and previously unknown mutational signatures (Islam *et al.* 2022). This type of analysis is predominately used for deriving reference signatures as it requires large cohorts of generally more than 100 samples. In contrast, *refitting* of mutational signatures is a numerical optimization approach that allows the assignment of known (in most cases, reference) signatures to an individual sample by quantifying the number of mutations attributed to each signature operative in that sample.

**Figure 1.**
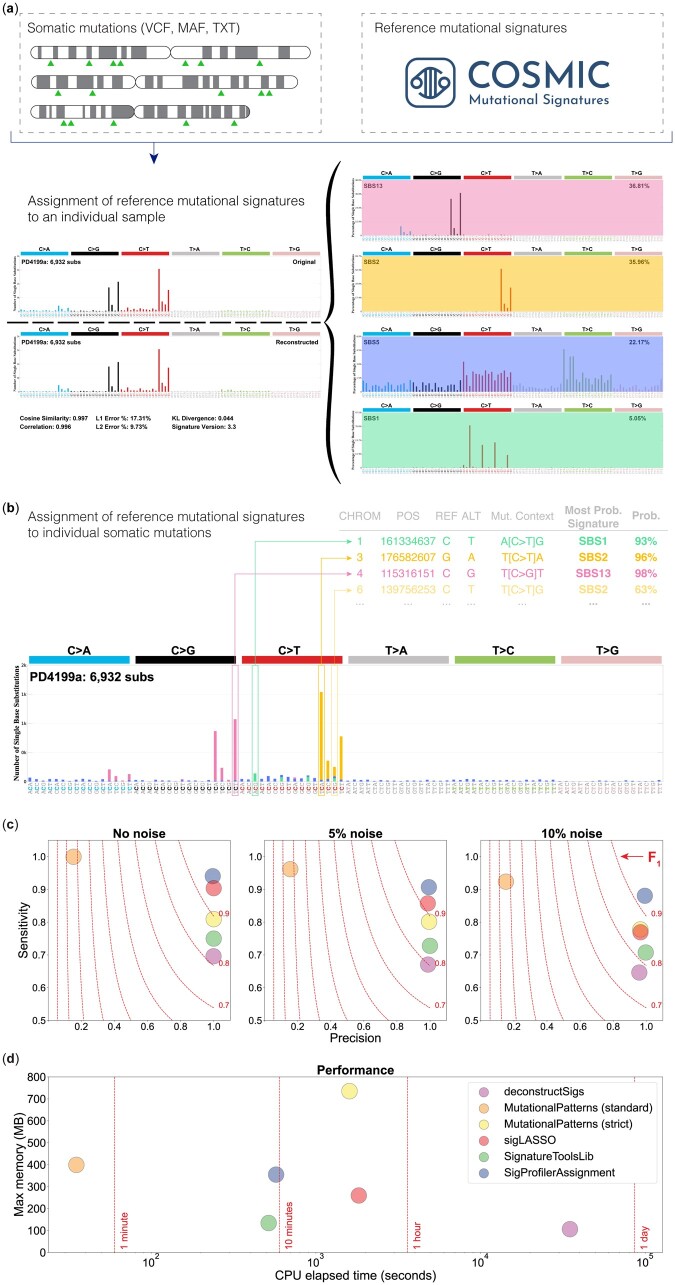
Assigning known mutational signatures to an individual sample and individual mutations with SigProfilerAssignment, and benchmarking with four other bioinformatics tools. SigProfilerAssignment supports input data in a standard format (VCF, MAF, or text) and it allows assigning a set of known signatures (e.g. ones from the COSMIC database) to an (a) individual sample and (b) probabilistically to an individual somatic mutation. Note that the probabilistic assignment of mutational signatures to an individual somatic mutation is only possible if a user provides a list of individual mutations (e.g. VCF file) for the examined sample instead of a mutational vector, as a mutational vector lacks information for individual mutations. (c) Accuracy benchmarking of SigProfilerAssignment and four other tools for assigning mutational signatures. Each tool was evaluated using 2700 synthetic cancer genomes generated using 21 different COSMIC reference mutational signatures. All COSMICv3.3 signatures were used as the input set of known mutational signatures. Three different levels of nonsystematic random noise (0%, 5%, and 10%) were used to evaluate the precision (*x*-axes), sensitivity (*y*-axes), and F_1_ scores (harmonic mean of precision and sensitivity; dotted lines) of each tool. (d) Computational benchmarking based on CPU elapsed time (*x*-axis; log-scaled) and maximum memory usage (*y*-axis) for each tool.

In the past decade, multiple tools for refitting known signatures were developed, including deconstructSigs (Rosenthal *et al.* 2016), MutationalPatterns (Blokzijl *et al.* 2018, Manders *et al.* 2022), sigLASSO (Li *et al.* 2020), and SignatureToolsLib (Degasperi *et al.* 2020, Degasperi *et al.* 2022). Most of these tools provide support almost exclusively for SBS signatures and lack an online interface, although a few web tools exist, including, MuSiCa (Díaz-Gay *et al.* 2018) and Mutalisk (Lee *et al.* 2018). Further, these tools have never been compared and no existing tool supports probabilistically assigning signatures to somatic mutations. To address these limitations, here, we present SigProfilerAssignment, a comprehensive bioinformatics tool for assigning mutational signatures to individual samples and individual somatic mutations (Fig. 1a and b). SigProfilerAssignment provides desktop and online support for all types of mutational signatures, including the COSMIC sets of reference SBS, DBS, ID, and CN signatures. Additionally, SigProfilerAssignment supports assignment of *de novo* extracted mutational signatures and of a user provided set of custom signatures. Our benchmarking based on 2700 simulated cancer genomes demonstrates that SigProfilerAssignment outperforms other commonly used tools on simulation data with and without noise.

## 2 Materials and methods

Given a set of known mutational signatures and a set of mutations in a cancer genome, both classified under the same mutational schema (Alexandrov *et al.* 2013, Bergstrom *et al.* 2019), SigProfilerAssignment identifies the number of mutations caused by each signature in that cancer genome (Fig. 1a). To quantity the number of mutations imprinted by each signature, SigProfilerAssignment uses a custom implementation of the forward stagewise algorithm (Hastie *et al.* 2009) and it applies nonnegative least squares (NNLS), based on the Lawson-Hanson method (Ling *et al.* 1977). The tool’s algorithm is available in Algorithm 1, and it is described in Supplementary Data. In addition to quantifying the activity of each mutational signature, SigProfilerAssignment also assigns known signatures to individual mutations (Fig. 1b) based on their specific mutational context.

Algorithm 1: Assigning mutational signatures to samples with SigProfilerAssignment

** Input:** $\vec{v}\;\in N_{+}^{\xi \times 1}$ (a vector corresponding to a set of mutations in a sample) and

     $S\in R_{+}^{\xi \times n}$ (a matrix corresponding to a set of n known mutational signatures)

** Output:** $\vec{a}\in N_{+}^{n\times 1}$ (the vector reflecting the activities of the n known signatures in sample $\vec{v})$

**1:** $\epsilon_{\min},\;\vec{a}=\;$ ***calcNNLS* (** $\vec{v},S$ **)**

   $S^{\mathrm{all}}=S$

**2: While** FLAG = True:

**3:  ** ** **$\epsilon_{\min},\;S\;$**= *removeSignatures*** ($\vec{v}$, $S,\epsilon_{\min}$)

**4:** $\epsilon_{\min},\;S\;$ **= *addSignatures*** ($\vec{v}$, $S^{\mathrm{all}},S,\;\epsilon_{\min}$)

**5:    Set** FLAG = False if S remains constant and there is no addition or removal of signatures

**    END While**

**6:** $\epsilon_{\min},\;\vec{a}\;=\;$ ***calcNNLS*(** $\vec{v},\;S$ **)**

**7: Return** $\;\vec{a}$

**8: FUNCTION removeSignatures** ($\vec{v}$, $S,\epsilon_{\min}$)

**9:   ** **While** FLAG = True:

**10:   ** ** For**j in **1** to size(S, 2) **do**         **//***loop from 1 to the total number of signatures in* S

**11:    ** ** **$\hat{S}=S[:,-j]$              **//***remove the j*^th^ signature from S

**12:    ** ** **$\epsilon [j],\;{\vec{a}}_{j}$**= *calcNNLS*(**$\vec{v},\;\hat{S}$**)**

** ** **     END For**

**13:   ** ** **$\mathrm{minIndex},\mathrm{minValue}=\min\left(\epsilon \right)$          **//***find the signature set with least relative error*

**14:   ** ** If (**$\mathrm{minValue}\;-\epsilon_{\min}\leq 0.01)\;$

**15:  **  ** **S= S[:,-minIndex]

** ** **     else**

**16:     Return** minValue, S 

** **     ** END If**

**    ** **END While**

** END removeSignatures**

**17: FUNCTION addSignatures** ($\vec{v}$, $S^{\mathrm{all}},S,\;\epsilon_{\min}$)

**18:  ** **While** FLAG = True:

**19:   ** ** For**p in **1** to size($S^{\mathrm{all}}$, 2) **do**          **//***loop from 1 to the total number of signatures in* $S^{\mathrm{all}}$

**20:    ** ** **$\hat{S}=\left[S;S^{\mathrm{all}}\left[:,p\right]\right]$              **//***add the p*^th^ signature from $S^{\mathrm{all}}$

**21:   ** **  **$\epsilon [j],\;{\vec{a}}_{j}$**= *calcNNLS*(**$\vec{v},\;\hat{S}$**)**

** ** **     END For**

**22: ** **   **$\mathrm{minIndex},\mathrm{minValue}=\min\left(\epsilon \right)\;\;\;\;\;\;\;\;\;$**//***find the signature set with least relative error*

**23: ** **   If (**$\epsilon_{\min}-\mathrm{minValue}\geq 0.05)\;$

**24: ** **    **$S=\;\left[S;S^{\mathrm{all}}\left[:,\mathrm{minValue}\right]\right]$

** ** **     else**

**25: ** **       Return**minValue, S 

** **     ** END If**

** **    **END While**

** END addSignatures**

**26: FUNCTION calcNNLS(** $\vec{v},\;S$ **)**

**27:** $\vec{a}\;=\;\mathrm{nnls}(S,\;\vec{v})$ **//***Calculating NNLS with the Lawson-Hanson method*

**28:** $\epsilon ={\left||\vec{v\;}-S\vec{a}|\right|}_{2}^{2}/{\left||\vec{v}|\right|}_{2}^{2}$ **//***Computing relative error*

**29:    Return** $\epsilon ,\;\vec{a}$

** END calcNNLS**

## 3 Results

To evaluate the performance of SigProfilerAssignment and another four commonly used tools for refitting mutational signatures (Rosenthal *et al.* 2016, Blokzijl *et al.* 2018, Degasperi *et al.* 2020, Li *et al.* 2020, Degasperi *et al.* 2022, Manders *et al.* 2022), we performed a comparative benchmarking using a previously generated independent synthetic dataset (Islam *et al.* 2022) (Fig. 1c and d). The dataset encompasses the SBS patterns of 2700 simulated cancer genomes, corresponding to 300 tumors from 9 different cancer types, generated using 21 different COSMIC reference signatures. To emulate a typical refitting of mutational signatures, the complete set of 79 COSMICv3.3 SBS signatures was used as input. The mutational signatures’ activities obtained by each tool were compared against the ground truth activities used to synthetically generate these samples. Three different levels of random noise (0%, 5%, and 10%) were tested to assess the robustness of the different algorithms in a real biological context. To evaluate the accuracy of the signature refitting, we calculated sensitivity, specificity, and F_1_ score (Supplementary Data). In addition, we also examined the runtime and memory utilization of each tool.

Our synthetic benchmarking revealed that SigProfilerAssignment outperforms all other approaches for the examined noise levels (Fig. 1c). For 10% random noise, only SigProfilerAssignment obtained an F1 score >0.90. In all cases, SigProfilerAssignment exhibited a high precision while showing an improved sensitivity compared to other approaches (Fig. 1c), with consistent top performance across cancer types (Supplementary Fig. S1) and most mutational signatures (Supplementary Fig. S2). In terms of computational performance, SigProfilerAssignment processed the 2700 samples within 9.6 min (0.21 s per sample; Fig. 1d). Only the standard mode of MutationalPatterns generated results substantially faster. However, MutationalPatterns’ standard mode exhibited sub-optimal performance, with a significant drop in precision for all noise levels, likely due to overfitting of the input data (Fig. 1c) (Blokzijl *et al.* 2018). This issue has been addressed in the most recent version of MutationalPatterns with the addition of a strict mode (Manders *et al.* 2022), albeit with a significant computational performance cost (Fig. 1d). Other approaches limit overfitting by implementing different penalties based on the L1 error (viz. sigLASSO) (Li *et al.* 2020) or the sum-squared error (viz. deconstructSigs) (Rosenthal *et al.* 2016), and post-hoc filters based on the percentage of the total number of mutations attributed to a given signature (viz. deconstructSigs and SignatureToolsLib) (Rosenthal *et al.* 2016, Degasperi *et al.* 2022) (Supplementary Table S1). No significant memory requirements were observed for any of the tools (Fig. 1d). Analysis of similar benchmarking datasets for DBS, ID, and CN mutational signatures (Supplementary Data) revealed that SigProfilerAssignment exhibits high precision and sensitivity, with F_1_ scores >0.85 for all assessed noise levels (Supplementary Fig. S3).

## 4 Conclusion

Assigning mutational signatures to individual samples provides an opportunity to identify the processes responsible for somatic mutations on a sample-by-sample basis. Considering our synthetic benchmarking, SigProfilerAssignment stands out as the most precise and sensitive tool while maintaining high computational performance and bringing novel capabilities. To the best of our knowledge, SigProfilerAssignment represents the first computational tool for assigning signature probabilities to individual mutations, which can allow uncovering the mutational processes responsible for specific driver genomic alterations leading to tumor evolution. SigProfilerAssignment is also the first tool that supports assignment of the recently developed copy number signatures (Steele *et al.* 2022), which are good predictors of clinical survival (Drews *et al.* 2022, Steele *et al.* 2022).

In summary, SigProfilerAssignment provides a novel computational package and an accessible online interface to accurately assign known mutational signatures to an individual cancer and individual somatic mutations, thus, enabling users to ascertain the mutational processes operative in a cancer genome.

## Supplementary Material

btad756_Supplementary_Data

## Data Availability

The data Data availability statement can be found in the supplementary materials.
