# Supplementary material for: Assigning mutational signatures to individual samples and individual somatic mutations with SigProfilerAssignment
Source: Bioinformatics. 2023 Dec 14;39(12):btad756. doi: 10.1093/bioinformatics/btad756 (PMC10746860; doi:10.1093/bioinformatics/btad756)
Supplement: btad756_Supplementary_Data [file btad756_supplementary_data.pdf]

SUPPLEMENTARY DATA

Description of SigProfilerAssignment's algorithm

Mathematically, a mutational schema can be represented as a finite alphabet Ξ of mutation types containing a total of ξ letters. Here, a mutational signature is defined as a probability mass function with domain the alphabet Ξ. In vector notations, a mutational signature can be denoted as $\vec{\boldsymbol{s}} = [s_1, s_2, \dots, s_\xi]^{\mathrm{T}}$, where $s_k, 1 \le k \le \xi$, is the probability for the mutational signature, $\vec{\boldsymbol{s}}$, to cause mutations of type corresponding to the kth letter of the alphabet Ξ. Since a mutational signature is a probability mass function, $0 \le s_k \le 1$ and $\sum_{k=1}^{\xi} s_k = 1$. As such, a set of known n mutational signatures can be expressed as a signature matrix, $\boldsymbol{S} \in \mathbb{R}_{+}^{\xi \times n}$, where $\boldsymbol{S} = [\vec{\boldsymbol{s}}^{\mathbf{1}}, \vec{\boldsymbol{s}}^{\mathbf{2}}, \dots, \vec{\boldsymbol{s}}^{\boldsymbol{n}}]$. Further, a set of mutations in a cancer genome can be defined as $\boldsymbol{v}\colon \Xi \to \mathbb{N}_{+}^{\xi}$. In vector notations, a set of mutations in a cancer genome $\vec{\boldsymbol{v}} = [v_1, v_2, \dots, v_\xi]^{\mathrm{T}}$, where $v_k, 1 \le k \le \xi$, reflects the number of mutations in that cancer genome of the mutation type corresponding to the kth letter of the alphabet Ξ. SigProfilerAssignment takes as an input a signature matrix, $\boldsymbol{S}$, and a set of mutations, $\vec{\boldsymbol{v}}$, to output a column vector of activities $\vec{\boldsymbol{a}} = [a_1, a_2, \dots, a_n]^{\mathrm{T}}$, where $a_t \in \mathbb{N}_{\mathbf{0}}^{n}, 1 \le t \le n$, corresponding to the number of somatic mutations attributed to the tth mutational signature. The underlying assumption of assigning mutational signatures is that the mutations within a sample can be approximated as a superposition of known mutational signatures and their activities:

$$\vec{\boldsymbol{v}} \approx \boldsymbol{S}\vec{\boldsymbol{a}} \qquad (1)$$

Thus, subject to $\vec{\boldsymbol{a}} \ge 0$, one needs to derive the vector $\vec{\boldsymbol{a}}$ that best fits the provided input data. To solve this optimization problem, SigProfilerAssignment uses a custom implementation of the forward stagewise algorithm (Hastie, et al., 2009) and it applies nonnegative least squares (NNLS) (Lawson and Hanson, 1977), based on the Lawson-Hanson method (Lawson and Hanson, 1977):

$$\min_{\vec{a} \geq 0} ||\vec{v} - S\vec{a}||_2^2 \quad (2)$$

The algorithm starts by first computing a minimum relative error, $\epsilon_{\min} = \frac{||\vec{v} - S\vec{a}||_2^2}{||\vec{v}||_2^2}$, by deriving the optimal nonnegative vector $\vec{a}$ for the complete set of all reference signatures, S, using equation *(2)*. This minimum error provides the best possible explanation of the data, but it also results in overfitting as all available signatures are utilized. Next, the tool uses steps for removing and adding signatures based on the backward and forward stepwise algorithms, respectively (Hastie, et al., 2009). First, signatures are removed by employing a backward stepwise algorithm (Hastie, et al., 2009) (**Algorithm 1**). Specifically, each signature from the reference signature set, S, is removed iteratively and the remaining signature set, $\hat{S}$, is attributed to the sample $\vec{v}$ by applying equation *(2)*. The increase in the relative error, $\epsilon_j = \frac{||\vec{v} - \hat{S}\vec{a}||_2^2}{||\vec{v}||_2^2} - \epsilon_{\min}$, due to removing a signature is calculated by removing the jth signature from S. The signature with the least relative increase in error rate is removed from the signature set, S, provided that the increase is less than a specific threshold (default value of 0.01). After the final removal of the signature with least relative error rate increase, the minimum relative error, $\epsilon_{\min}$, and the set of signatures, S, are updated to reflect this removal. The removal step is repeated until all signatures satisfying the conditions are removed from S. The removal steps are followed by addition steps based on the forward stepwise algorithm (Hastie, et al., 2009) (**Algorithm 1**). Specifically, each of the previously removed reference signatures is added back iteratively to S and the new signature set, $\hat{S}$, is fit for the sample $\vec{v}$ by applying equation *(2)*. Thus, the decrease in the relative error, $\epsilon_l = \epsilon_{\min} - \frac{||\vec{v} - \hat{S}\vec{a}||_2^2}{||\vec{v}||_2^2}$, due to adding a signature is calculated by adding the lth signature to S. The signature with maximum relative decrease of the error rate is added back to the signature set, S, provided that the increase is more than a specific threshold (default value of 0.05). After the final addition of the signature with most

relative rate decrease, the minimum relative error, $\epsilon_{\min}$, and the set of signatures, $\boldsymbol{S}$, are updated to reflect this addition. The addition step is repeated until all signatures satisfying the conditions are added back to $\boldsymbol{S}$. Lastly, the addition and removal steps are repeated until convergence, where no signature is added or removed from the list of signatures (**Algorithm 1**).

In addition to quantifying the activity of each mutational signature, SigProfilerAssignment also assigns known signatures to individual mutations (**Fig. 1*B***) based on their specific mutational context:

$$p_k^t = \frac{s_k^t \, a_t}{[\boldsymbol{S}\vec{\boldsymbol{a}}]_k} \tag{3}$$

where, p_k^t represents the probability of a mutation corresponding to the kth letter of the alphabet Ξ being caused by the tth signature in the sample; s_k^t is the probability of the tth signature to cause mutation corresponding to the kth letter of the alphabet Ξ; a_t is the number of mutations attributed to the tth mutational signature; and $[\boldsymbol{S}\vec{\boldsymbol{a}}]_k$ is the value of the kth element of the vector obtained by the matrix multiplication of the signature matrix, $\boldsymbol{S}$, and the derived signature activities, $\vec{\boldsymbol{a}}$.

Distribution and Usage

SigProfilerAssignment is distributed as a Python package and it is available under a permissive BSD 2-clause license at https://github.com/AlexandrovLab/SigProfilerAssignment and https://pypi.org/project/SigProfilerAssignment/. An R wrapper is also provided using the same license at https://github.com/AlexandrovLab/SigProfilerAssignmentR. SigProfilerAssignment provides support for most operating systems, including Windows, macOS, and Linux-based systems, and has an extensive documentation at https://osf.io/mz79v/wiki/home/. In addition, a user-friendly online interface is provided as part of the COSMIC Mutational Signatures website

(Tate, et al., 2019) at https://cancer.sanger.ac.uk/signatures/assignment/. For compliance with EU and UK specific privacy regulations, the COSMIC website requires free registration prior to using SigProfilerAssignment. This ensures that all uploaded user data are maintained privately and purged properly.

Input data for both desktop and online versions can be provided by mutation calling and segmentation files, depending on the variant class, and is processed internally by SigProfilerMatrixGenerator (Bergstrom, et al., 2019; Khandekar, et al., 2023). The tool supports common formats for SBS, DBS, and ID somatic mutations, including the Variant Call Format (VCF), the Mutation Annotation Format (MAF), and simple text files. Multi-sample segmentation files obtained from ASCAT (Van Loo, et al., 2010), ABSOLUTE (Carter, et al., 2012), Sequenza (Favero, et al., 2015), FACETS (Shen and Seshan, 2016), Battenberg (Van Loo, et al., 2010), or PURPLE (Shale, et al., 2022) are supported for analysis of copy number signatures. In addition, SigProfilerAssignment can use standard mutational matrices, where rows correspond to mutational channels and columns to samples, extracted from the SigProfiler suite of tools (Bergstrom, et al., 2019; Bergstrom, et al., 2022; Islam, et al., 2022). Different sequencing assays (whole genome sequencing, whole exome sequencing, and targeted sequencing), species (human, mouse, and rat), genome builds (GRCh37/38, mm9/10, and rn6), and signatures (default COSMICv3.3 (Tate, et al., 2019), prior COSMIC versions, and custom signature databases) are supported.

The main output of SigProfilerAssignment includes the activity of each known mutational signature for each of the supplied samples, the reconstruction of the original dataset, and the probability of each individual mutation being caused by a specific signature. The latter is not

provided when the input file is a mutational vector or mutational matrix as this input format lacks information about individual somatic mutations. Signature activities correspond to the specific numbers of mutations from the original catalog caused by a particular mutational process. Considering these activities, as well as the provided set of known mutational signatures, a reconstruction of the original mutational catalog for each sample is derived. Different accuracy metrics for this reconstruction are outputted by SigProfilerAssignment, including cosine similarity, Kullback–Leibler divergence, Pearson correlation, L1 relative error, and L2 relative error.

The signature assignment results are summarized using three independent visualizations: *(i)* a bar plot depicting the activities of all mutational signatures within a sample; *(ii)* a tumor mutational burden (TMB) signature plot showing the activities per mutational signature; and *(iii)* an individual reconstruction plot per sample, which includes the mutational profiles for both the original and the reconstructed input sample, different accuracy metrics, and the mutational profiles for each of the known mutational signatures assigned to that sample. For the online version of the tool, an interactive heatmap plot, including the signatures' activities and the samples' reconstruction accuracies is also provided. Raw data files containing activities, reconstruction metrics, and signature probabilities for individual mutations are generated by the desktop tool and can be downloaded from the online version.

Benchmarking of bioinformatics tools for refitting known mutational signatures

To evaluate the performance of tools for refitting known mutational signature, we used a standard set of evaluation metrics and compared SigProfilerAssignment with another four commonly used approaches: deconstructSigs (Rosenthal, et al., 2016), MutationalPatterns (Blokzijl, et al., 2018;

Manders, et al., 2022), sigLASSO (Li, et al., 2020), and SignatureToolsLib (Degasperi, et al., 2020; Degasperi, et al., 2022). Specifically, each tool was applied to 2,700 previously simulated cancer genomes (Islam, et al., 2022), corresponding to 300 simulated tumors from nine different cancer types, including: bladder transitional cell carcinoma, esophageal adenocarcinoma, breast adenocarcinoma, lung squamous cell carcinoma, renal cell carcinoma, ovarian adenocarcinoma, osteosarcoma, cervical adenocarcinoma, and stomach adenocarcinoma. The cancer genomes of these samples were simulated using 21 different COSMIC SBS reference signatures. To emulate a typical refitting of mutational signatures, each tool was applied by utilizing the complete set of 79 COSMICv3.3 SBS signatures. After assigning the signatures, the assignment of each signature to each sample was classified as either a *true positive* (TP), *false positive* (FP), or *false negative* (FN) result. A known signature was considered TP if at least one mutation was assigned to the signature by a particular tool and the ground truth activity of the signature was greater than zero. In contrast, a signature was classified as FP when it was assigned by a tool, but the ground truth activity was zero. Lastly, FN results were signatures with ground truth activities above zero that were not assigned any somatic mutation. These standard metrics allowed calculating the precision, sensitivity, and F_1 score of each tool per sample, defined as:

$$Precision = \frac{TP}{TP + FP}$$

$$Sensitivity = \frac{TP}{TP + FN}$$

$$F_1\, score = 2 * \frac{Precision\ *\ Sensitivity}{Precision\ +\ Sensitivity}$$

These metrics were calculated for each synthetically generated sample and, subsequently, averaged to obtain a final accuracy value for each random noise level (0%, 5%, and 10%).

For the ID and DBS benchmarking, synthetic mutational profiles were generated following the same methodology used for constructing the previously published SBS dataset (Islam, et al., 2022), using the GenerateSyntheticTumors function of the SynSigGen R package (https://github.com/steverozen/SynSigGen). This package uses the original activities from the PCAWG analysis of mutational signatures (Alexandrov, et al., 2020) to derive synthetic mutational profiles per cancer type. This simulation process requires that at least two different signatures are assigned to each sample from every specific cancer type. Considering this, we generated synthetic datasets for DBS and ID variant classes using the same nine cancer types previously used in the SBS benchmarking (300 simulated samples from each cancer type), including bladder transitional cell carcinoma, esophageal adenocarcinoma, breast adenocarcinoma, lung squamous cell carcinoma, renal cell carcinoma, ovarian adenocarcinoma, osteosarcoma, cervical adenocarcinoma, and stomach adenocarcinoma. However, due to the limitation mentioned above, cervical adenocarcinoma was removed for the synthetic ID profile generation since only ID1 was present in the original activities of the PCAWG samples. For the generation of the synthetic DBS dataset, cervical adenocarcinoma was also removed (only DBS4 was assigned to one of the PCAWG samples), along with lung squamous cell carcinoma, as only the tobacco-associated DBS2 signature was assigned to several of the PCAWG cases. In summary, 2,100 synthetic DBS samples and 2,400 synthetic ID samples were generated (300 for each of the seven and eight cancer types, respectively). In the case of copy number alterations, since this mutation type was not supported by SynSigGen, the pan-cancer activities from the original publication describing the COSMICv3.3 CN signatures (Steele, et al., 2022) were used and multiplied by the reference signatures to get a synthetic dataset encompassing 9,699 synthetic samples from 33 different

cancer types. Regarding the input set of known mutational signatures, in all three cases the most recent COSMICv3.3 version of the reference signatures was used, including 18 ID, 11 DBS, and 24 CN signatures.

To benchmark the computational performance of the different bioinformatics tools, their CPU elapsed time and peak memory usage were monitored and averaged for the three noise levels.

SigProfilerAssignment v0.0.28 was run using default parameters. deconstructSigs (Rosenthal, et al., 2016) v1.8.0 was used with default parameters as indicated in https://github.com/raerose01/deconstructSigs/. MutationalPatterns (Manders, et al., 2022) v3.0.1 was run with default parameters independently using its standard and strict modes, corresponding to the *fit_to_signatures* and *fit_to_signatures_strict* functions, respectively. The *max_delta* parameter was fixed to a default value of 0.004 for the strict mode, according to authors' instructions at https://bioconductor.org/packages/release/bioc/vignettes/MutationalPatterns/inst/doc/Introduction_to_MutationalPatterns.html. sigLASSO (Li, et al., 2020) v1.1 was used with default parameters (no priors) following the instructions at https://github.com/gersteinlab/siglasso; albeit avoiding the generation of plots for the comparison of the computational performance. SignatureToolsLib (Degasperi, et al., 2022) v2.1.2 was run with global signatures using the *Fit* function and default parameters as indicated at https://github.com/Nik-Zainal-Group/signature.tools.lib.

Data availability

All synthetic benchmarking data used in this article are available on FigShare at https://doi.org/10.6084/m9.figshare.24457114 and the SBS benchmarking data were and the SBS benchmarking data were originally generated as part of (Islam, et al., 2022). They are publicly available under the Creative Commons Attribution 4.0 International license. SigProfilerAssignment is developed as a Python package and it is available under a permissive BSD 2-clause license at https://github.com/AlexandrovLab/SigProfilerAssignment and https://pypi.org/project/SigProfilerAssignment/. An R wrapper is also provided using the same license at https://github.com/AlexandrovLab/SigProfilerAssignmentR. SigProfilerAssignment provides support for most operating systems, including Windows, macOS, and Linux-based systems. An online version of the tool, requiring a free registration, is provided as part of the COSMIC Mutational Signatures website at https://cancer.sanger.ac.uk/signatures/assignment/.

SUPPLEMENTARY TABLES

Tool name	Input data (mutations)	Platform	Optimization Method	Algorithm	Computational engine	Penalties	Post hoc filter (TMB threshold)
deconstructSigs (Rosenthal, et al., 2016)	matrix, custom	R	Multiple linear regression with a nonnegative cutoff on activities	Golden-section search algorithm (Kiefer, 1953)	Original implementation	Addition penalty (SSE; default: 0.001)	Yes (6%)
MutationalPatterns (standard) (Blokzijl, et al., 2018)	matrix, VCF	R	NNLS	Levenberg-Marquardt algorithm (Levenberg, 1944)	Pracma R package (Borchers, 2022)	No penalties	No
MutationalPatterns (strict) (Manders, et al., 2022)	matrix, VCF	R	NNLS	Levenberg-Marquardt algorithm (Levenberg, 1944)	Original implementation (penalty framework) and Pracma R package (Borchers, 2022)	Removal penalty (cosine similarity; default: 0.004)	No
sigLASSO (Li, et al., 2020)	matrix, VCF, MAF, custom	R	Non-negative linear LASSO regression	Alternative convex search algorithm (Gorski, et al., 2007)	Original implementation (framework) and glmnet R package (Lasso regression) (Friedman, et al., 2010)	Optimized penalty (L1 norm). Priors. Lambda hyperparameter.	No
SignatureToolsLib (Degasperi, et al., 2020; Degasperi, et al., 2022)	matrix, VCF, BEDPE, custom	R / Web app	Non-negative linear regression (KL Divergence objective function)	Lee's multiplicative algorithm (Lee and Seung, 1999)	NNLM R package (Lin and Boutros, 2020)	No penalties	Yes (5%)
SigProfilerAssignment	matrix, VCF, MAF, segmentation, custom	Python / R / Web app	NNLS	Lawson-Hanson algorithm (Lawson and Hanson, 1977)	Original implementation (penalty framework) and Scipy python package (NNLS) (Virtanen, et al., 2020)	Initial removal, addition, and removal penalties (L2 norm; default: 0.05, 0.05 and 0.01)	No

Table S1. Overview of bioinformatics tools for assignment of mutational signatures. Tools are ordered alphabetically. The table columns represent the following: benchmarking tool's name, supported input data types, compatible operational platforms, employed optimization method, primary fitting algorithm, computational engine utilized, enforced additional penalties, and tumor mutational burden percentage threshold to avoid overfitting signatures. MAF, mutation annotation

format; matrix: mutational matrix; NNLM, non-negative linear models; NNLS, non-negative least squares; SSE, sum of squared errors; TMB, tumor mutational burden; VCF, variant call format.

SUPPLEMENTARY FIGURES

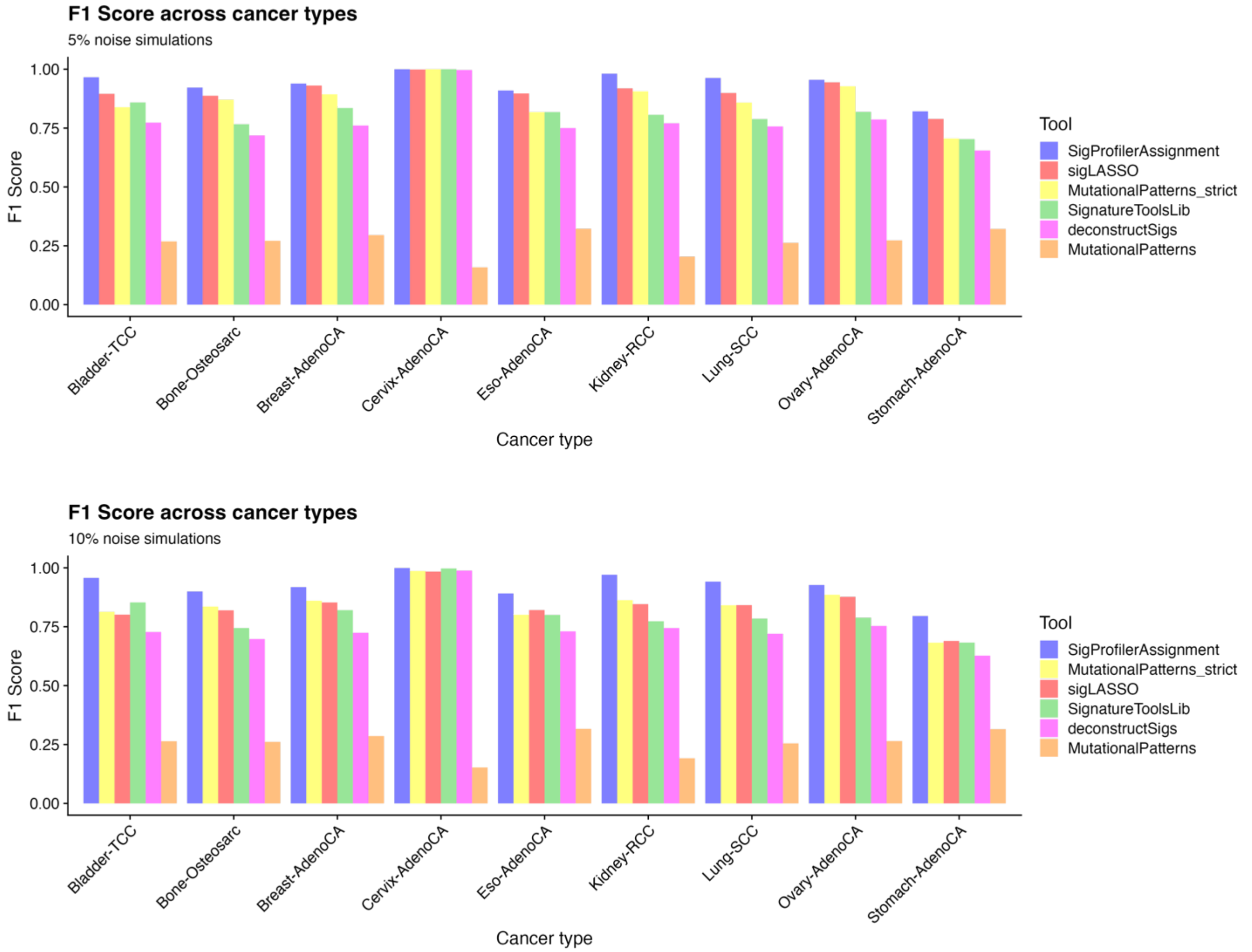

Figure S1. Tissue type-specific benchmarking of SigProfilerAssignment and four other tools for assigning mutational signatures. The F_1 scores (harmonic mean of precision and sensitivity) for the nine cancer types included in the synthetic dataset (300 simulated genomes for each cancer type) were used to evaluate the accuracy of the signature assignment across the simulations with non-systematic random noise.

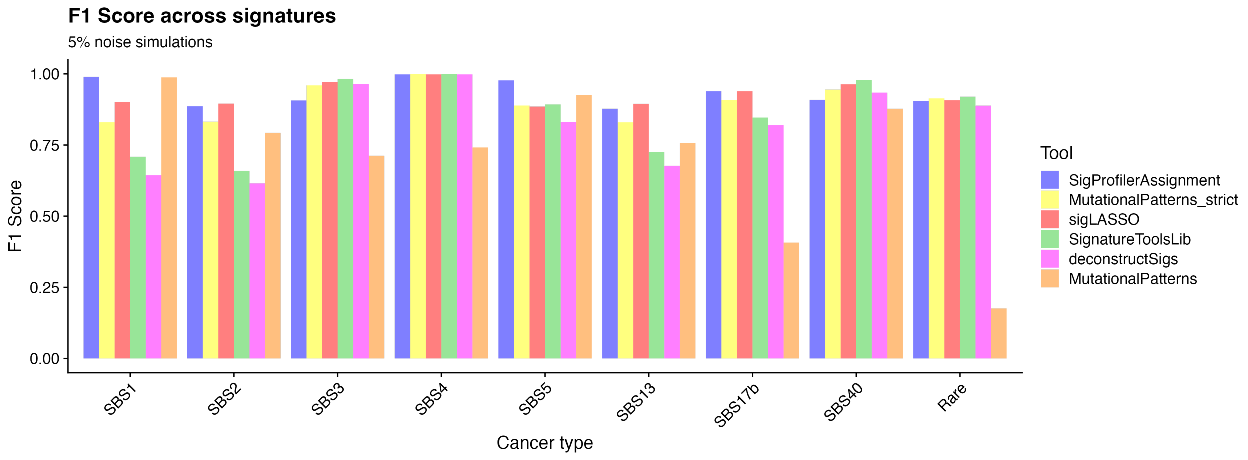

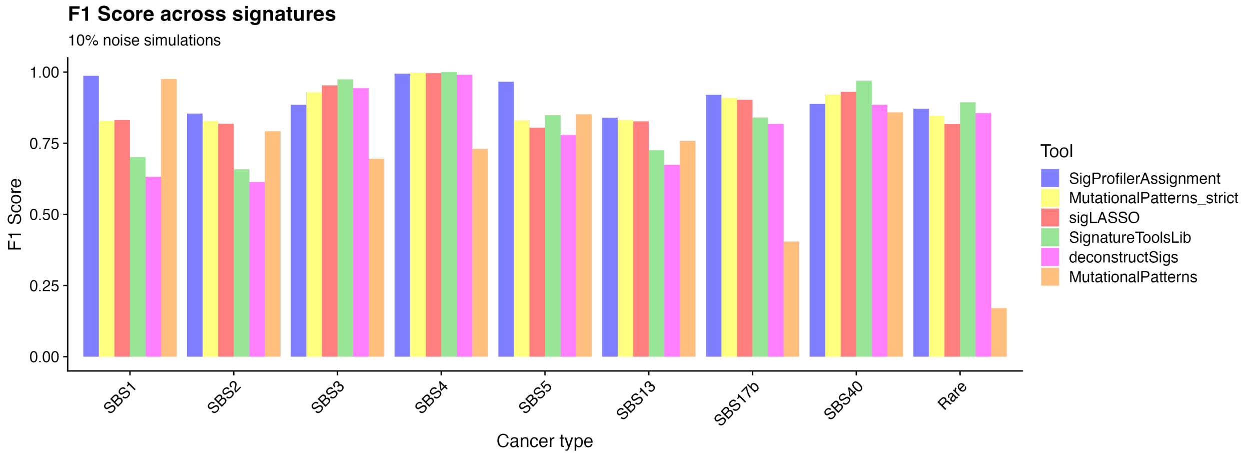

Figure S2. Signature-specific benchmarking of SigProfilerAssignment and four other tools for assigning mutational signatures. The F_1 scores (harmonic mean of precision and sensitivity) for the eight most prevalent mutational signatures across the ground truth activities and the average of the remaining 13 rare signatures were used to evaluate the accuracy of the signature assignment across the simulations with non-systematic random noise.

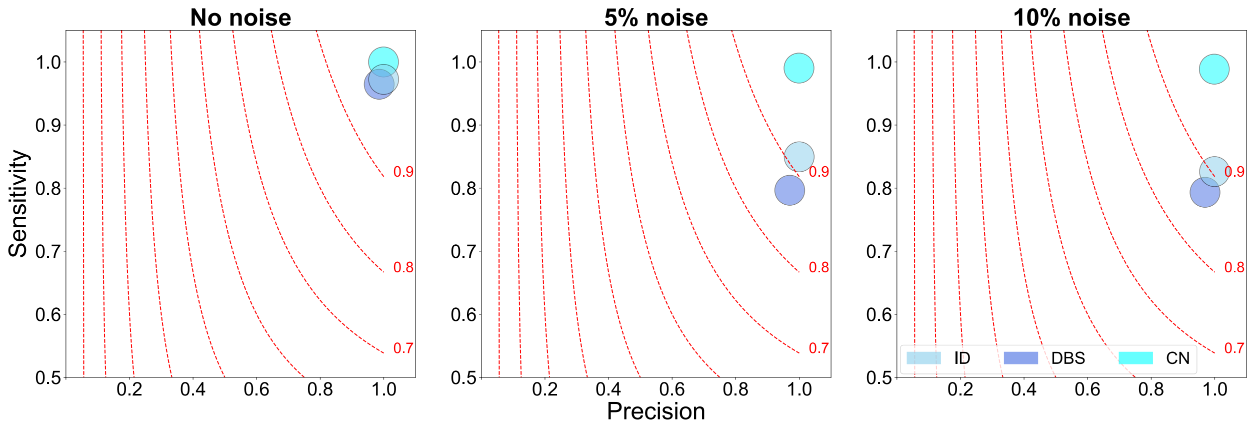

Figure S3. Benchmarking of SigProfilerAssignment across different mutation types. Synthetic DBS, ID, and CN samples were used to test the accuracy of SigProfilerAssignment signature assignment using COSMICv3.3 signatures as input known mutational signatures. Three different levels of non-systematic random noise (0%, 5%, and 10%) were used to evaluate the precision (x-axes), sensitivity (y-axes), and F_1 scores (harmonic mean of precision and sensitivity; red dotted lines) of each tool.

SUPPLEMENTARY REFERENCES

Alexandrov, L.B., *et al.* The repertoire of mutational signatures in human cancer. *Nature* 2020;578(7793):94-101.

Bergstrom, E.N., *et al.* SigProfilerMatrixGenerator: a tool for visualizing and exploring patterns of small mutational events. *BMC Genomics* 2019;20(1):685.

Bergstrom, E.N., *et al.* Examining clustered somatic mutations with SigProfilerClusters. *Bioinformatics* 2022;38(13):3470-3.

Blokzijl, F., *et al.* MutationalPatterns: comprehensive genome-wide analysis of mutational processes. *Genome Med* 2018;10(1):33.

Borchers, H.W. 2022. pracma: Practical Numerical Math Functions. (https://CRAN.R-project.org/package=pracma last accessed).

Carter, S.L., *et al.* Absolute quantification of somatic DNA alterations in human cancer. *Nat Biotechnol* 2012;30(5):413-21.

Degasperi, A., *et al.* A practical framework and online tool for mutational signature analyses show inter-tissue variation and driver dependencies. *Nat Cancer* 2020;1(2):249-63.

Degasperi, A., *et al.* Substitution mutational signatures in whole-genome–sequenced cancers in the UK population. *Science* 2022;376(6591).

Favero, F., *et al.* Sequenza: allele-specific copy number and mutation profiles from tumor sequencing data. *Ann Oncol* 2015;26(1):64-70.

Friedman, J.H., Hastie, T. and Tibshirani, R. Regularization Paths for Generalized Linear Models via Coordinate Descent. *Journal of Statistical Software* 2010;33(1):1 - 22.

Gorski, J., Pfeuffer, F. and Klamroth, K. Biconvex sets and optimization with biconvex functions: a survey and extensions. *Mathematical Methods of Operations Research* 2007;66(3):373-407.

Hastie, T., Tibshirani, R. and Friedman, J.H. The Elements of Statistical Learning: Data Mining, Inference, and Prediction. Springer; 2009.

Islam, S.M.A., *et al.* Uncovering novel mutational signatures by de novo extraction with SigProfilerExtractor. *Cell Genom* 2022;2(11):None.

Khandekar, A., *et al.* Visualizing and exploring patterns of large mutational events with SigProfilerMatrixGenerator. *bioRxiv* 2023:2023.02.03.527015.

Kiefer, J. Sequential minimax search for a maximum. *Proceedings of the American Mathematical Society* 1953;4(3):502–6.

Lawson, C.L. and Hanson, R.J. Solving Least Squares Problems. *Journal of the American Statistical Association* 1977;72(360):930-1.

Lee, D.D. and Seung, H.S. Learning the parts of objects by non-negative matrix factorization. *Nature* 1999;401(6755):788-91.

Levenberg, K. A METHOD FOR THE SOLUTION OF CERTAIN NON-LINEAR PROBLEMS IN LEAST SQUARES. *Quarterly of Applied Mathematics* 1944;2(2):164-8.

Li, S., Crawford, F.W. and Gerstein, M.B. Using sigLASSO to optimize cancer mutation signatures jointly with sampling likelihood. *Nature Communications* 2020;11(1):3575.

Lin, X. and Boutros, P.C. Optimization and expansion of non-negative matrix factorization. *BMC Bioinformatics* 2020;21(1):7.

Manders, F., *et al.* MutationalPatterns: the one stop shop for the analysis of mutational processes. *BMC Genomics* 2022;23(1):134.

Rosenthal, R., *et al.* DeconstructSigs: delineating mutational processes in single tumors distinguishes DNA repair deficiencies and patterns of carcinoma evolution. *Genome Biol* 2016;17:31.

Shale, C., *et al.* Unscrambling cancer genomes via integrated analysis of structural variation and copy number. *Cell Genomics* 2022;2(4):100112.

Shen, R. and Seshan, V.E. FACETS: allele-specific copy number and clonal heterogeneity analysis tool for high-throughput DNA sequencing. *Nucleic Acids Res* 2016;44(16):e131.

Steele, C.D., *et al.* Signatures of copy number alterations in human cancer. *Nature* 2022;606(7916):984-91.

Tate, J.G., *et al.* COSMIC: the Catalogue Of Somatic Mutations In Cancer. *Nucleic Acids Res* 2019;47(D1):D941-D7.

Van Loo, P., *et al.* Allele-specific copy number analysis of tumors. *Proc Natl Acad Sci U S A* 2010;107(39):16910-5.

Virtanen, P., *et al.* SciPy 1.0: fundamental algorithms for scientific computing in Python. *Nature Methods* 2020;17(3):261-72.
